# Molecular Mechanisms of HTLV-1 Cell-to-Cell Transmission

**DOI:** 10.3390/v8030074

**Published:** 2016-03-09

**Authors:** Christine Gross, Andrea K. Thoma-Kress

**Affiliations:** Institute of Clinical and Molecular Virology, Friedrich-Alexander-Universität Erlangen-Nürnberg (FAU), 91054 Erlangen, Germany; christine.gross@viro.med.uni-erlangen.de

**Keywords:** HTLV-1, Tax, p8, virus transmission, cell-to-cell transmission, cell-cell contacts, virological synapse, viral biofilm, cellular conduit

## Abstract

The tumorvirus human T-cell lymphotropic virus type 1 (HTLV-1), a member of the delta-retrovirus family, is transmitted via cell-containing body fluids such as blood products, semen, and breast milk. *In vivo*, HTLV-1 preferentially infects CD4^+^ T-cells, and to a lesser extent, CD8^+^ T-cells, dendritic cells, and monocytes. Efficient infection of CD4^+^ T-cells requires cell-cell contacts while cell-free virus transmission is inefficient. Two types of cell-cell contacts have been described to be critical for HTLV-1 transmission, tight junctions and cellular conduits. Further, two non-exclusive mechanisms of virus transmission at cell-cell contacts have been proposed: (1) polarized budding of HTLV-1 into synaptic clefts; and (2) cell surface transfer of viral biofilms at virological synapses. In contrast to CD4^+^ T-cells, dendritic cells can be infected cell-free and, to a greater extent, via viral biofilms *in vitro*. Cell-to-cell transmission of HTLV-1 requires a coordinated action of steps in the virus infectious cycle with events in the cell-cell adhesion process; therefore, virus propagation from cell-to-cell depends on specific interactions between cellular and viral proteins. Here, we review the molecular mechanisms of HTLV-1 transmission with a focus on the HTLV-1-encoded proteins Tax and p8, their impact on host cell factors mediating cell-cell contacts, cytoskeletal remodeling, and thus, virus propagation.

## 1. Introduction

Human T-cell lymphotropic virus type 1 (HTLV-1), a delta-retrovirus, is the causative agent of a severe and fatal lymphoproliferative disorder of CD4^+^ T-cells, adult T-cell leukemia/lymphoma (ATL), and of a neurodegenerative, inflammatory disease, HTLV-1-associated myelopathy/tropical spastic paraparesis (HAM/TSP) [1,2,3,4,5]. Up to 5% of infected people develop one of the aforementioned diseases as a consequence of prolonged viral persistence after a clinical latency period that may last over decades [6,7,8]. Although the exact number of infected people is unknown [9], it is estimated that 5–10 million people worldwide are infected with HTLV-1 [10]. Endemic regions for HTLV-1 are Japan, Melanesia, South America, parts of sub-Saharan Africa, the Caribbean, central parts of Australia, and the Middle East [10,11]. In Europe, only Romania seems to be an endemic region [10,12,13].

Upon binding to its receptor, which is composed of the widely expressed glucose transporter 1 (Glut-1), neuropilin-1 (NRP-1, BDCA-4), and heparan sulfate proteoglycans (HSPG), HTLV-1 enters and infects its target cell [14,15,16,17,18]. After uncoating and reverse transcription, HTLV-1 integrates into the host cell genome and is predominantly maintained in its provirus form (9.1 kb), which is flanked by long terminal repeats (LTR) in both the 5' and 3' region carrying the viral promoter (reviewed by [19]). Next to genes common for retroviruses encoding structural proteins Gag, the enzymes protease, polymerase, integrase, and reverse transcriptase, HTLV-1 encodes regulatory (Tax, Rex) and accessory (p12/p8, p13, p30) proteins from the sense strand and the HTLV-1 basic leucine zipper (HBZ) from the antisense strand [7,19]. Tax and Rex are essential for viral replication. While Tax enhances viral mRNA synthesis by transactivating the HTLV-1 promoter located in the 5'-LTR, Rex controls the synthesis of the structural proteins on a post-transcriptional level [20]. The accessory proteins p12/p8, p13, and p30 are important for viral infectivity and persistence *in vivo*, but not for virus replication *in vitro* [21]. An important role in HTLV-1-induced cellular transformation has been attributed to the viral oncoprotein Tax, which is sufficient to immortalize T-cells *in vitro* [19]. During the last decade, important roles for promoting viral replication and cellular proliferation have been attributed to the HBZ protein and to HBZ RNA. It is thought that Tax is important for initiating immortalization of lymphocytes, while HBZ is essential for maintaining the immortalized phenotype [22].

HTLV-1 replicates either by infecting new target cells or by mitotic division and clonal proliferation of infected cells (for review see [23]). In this article, we review the molecular mechanisms of infectious HTLV-1 cell-to-cell transmission. We focus on the HTLV-1-encoded proteins Tax and p8, their impact on host factors mediating cell-cell contacts, cytoskeletal remodeling, and virus transmission.

## 2. Target Cells of HTLV-1 *in Vivo*

While HTLV-1 infects several cell types *in vitro* after binding of the viral envelope (Env) protein to the HTLV-1 receptor [14,15,16,17,18], CD4^+^ T-cells are the main and preferential target for HTLV-1 infection *in vivo* [24]. Additionally, HTLV-1 proviral DNA can also be detected to a lesser extent in CD8^+^ T-cells [25,26,27], dendritic cells (DC) [28], plasmacytoid dendritic cells (pDC) [29], and monocytes [26,30]. A recent study by Melamed *et al.* has shown that infected CD8^+^ T-cells constitute about 5% of the total HTLV-1 proviral load found in peripheral blood mononuclear cells (PBMC) in a cohort of 12 HTLV-1-infected patients [27]. However, in clonally expanded populations of HTLV-1-infected cells, it seems unlikely that other cell types than CD4^+^ and CD8^+^ cells are present because almost all (99.7%) of the most highly abundant clones were CD4^+^ or CD8^+^ cells [27]. Another recent study reported the presence of HTLV-1 in classical, intermediate, and non-classical monocytes in PBMC of HTLV-1-infected individuals. HTLV-1 infection altered surface receptor expression, migratory function, and subset frequency of the monocytes [31]. The authors proposed the model that recruitment of classical monocytes to inflammation sites is increased in infected patients, which may result in virus acquisition and enhanced virus dissemination [30]. These *ex vivo* observations are in contrast to *in vitro* observations showing that monocytes are refractory to productive HTLV-1 infection, which initiates Caspase-3-dependent cell death [32]. Early work has also shown that HTLV-1-infected B-cell clones can be isolated from ATL patients and that B-cells are targets of HTLV-1 *in vitro* [31,33,34,35,36]. However, B-cells do not seem to constitute a major viral reservoir *in vivo*.

## 3. Routes of Viral Transmission *in Vivo*

In contrast to human immunodeficiency virus (HIV), cell-free infection of CD4^+^ T-cells with HTLV-1 is very inefficient. Free virions can hardly be detected in the blood plasma of infected individuals and are poorly infectious for most cell types except DC [37,38,39,40,41]. Further, infected lymphocytes produce a limited amount of viral particles, amongst which 1 out of 10^5^ is infectious [38]. However, transmission is greatly improved upon establishment of cell-cell contacts [40]. Therefore, efficient virus transmission occurs via cell-containing body fluids such as blood, semen, and breast milk (for review, see [40]). In endemic regions, HTLV-1 is primarily transmitted from mother to child. Contrary to HIV, mother-to-child transmission of HTLV-1 predominantly occurs via breast-feeding, while transplacental transmission or transmission during delivery are rare [40,42,43]. The risk of viral transmission increases with longer breast-feeding periods and high maternal proviral load. Reduction in breast-feeding also reduces mother-to-child transmission [44]. Sexual transmission of HTLV-1 occurs more efficiently from men to women than *vice versa* [40], and transmission might be enhanced by other sexually transmitted diseases that cause ulcers and ruptures of the mucosa like syphilis or Herpes simplex type 2 [45]. Rarely, HTLV-1 can also be transmitted by organ transplantation and cause diseases in immunocompromised transplant recipients, like HTLV-1-associated lymphomas or HAM/TSP after kidney transplantation [46,47]. HTLV-1 is not only transmittable among humans, but also from non-human primates (NHP) to humans. Recent studies have reported that interspecies transmission of the simian counterpart STLV-1 through severe bites from NHP is an ongoing event in Central Africa [48,49].

It is still not settled whether cell-free or cell-associated HTLV-1 accounts for infectivity of the primary target cell *in vivo*. Moreover, the first host cell infected by HTLV-1 *in vivo* and the exact route of infection are currently unknown [50]. Since antigen-presenting cells such as DC (see Section 5) are naturally infected with HTLV-1, it is assumed that they could be involved in viral transmission to T-cells *in vivo*. To obtain insights into the first steps of HTLV-1 acquisition *in vivo*, e.g., during mother-to-child transmission by breastfeeding, Martin-Ladil *et al.* developed an *in vitro* model studying the transcytosis of HTLV-1 across a barrier of enterocytes [51]. Interestingly, the integrity of the epithelial barrier was maintained during co-culture with HTLV-1-infected lymphocytes, and enterocytes were not susceptible to HTLV-1 infection. However, free infectious HTLV-1 virions crossed the epithelial barrier via transcytosis and productively infected human DC located beneath the epithelial barrier [51]. Upon infection, DC could then pass the virus to T-cells. Surprisingly, DC are more susceptible to *in vitro* infection with viral biofilms than autologous CD4^+^ T-cells, underlining their potential importance in virus dissemination [50].

The study of HTLV-1 infection *in vivo* has benefitted from small animal models (rabbits, rats, and mice) and from large animal models (macaques, sheep infected with the related bovine leukemia virus) [52,53]. Recently, HTLV-1-infected humanized mice that are reconstituted with a functional human immune system and that develop lymphomas have been described [54]. Humanized mice may provide the opportunity to visualize HTLV-1 transmission *in vivo* as it has been shown for transmission of the related retroviruses murine leukemia virus (MLV) and HIV [55]. Additionally, humanized mouse models have already been used to show the neutralizing function of anti-Env antibodies in preventing HTLV-1 transmission *in vivo* [56].

## 4. Molecular Mechanisms of HTLV-1 Cell-to-Cell Transmission between CD4^+^ T-Cells

Cell-cell-mediated virus propagation requires coordination of steps of the virus infectious cycle with events in the cell-cell adhesion process. Therefore, the mechanism of cell-to-cell transmission depends on specific interactions between cellular and viral proteins [57]. Thus far, two types of cell-cell contacts have been described to be critical for HTLV-1 transmission, tight cell-cell contacts (see Section 4.1) and cellular conduits (see Section 4.2). For transmission at tight cell-cell contacts, two non-exclusive mechanisms of virus transmission at the virological synapse (VS) have been proposed: (1) polarized budding of HTLV-1 into synaptic clefts (see Section 4.1.1), and (2) cell surface transfer of viral biofilms (see Section 4.1.2). Thus far, the mechanism of HTLV-1 transmission via cellular conduits induced by the viral p8 protein is unclear (see Section 4.2) [58,59]. Independent of the route of HTLV-1 transmission, viral particles are transmitted in confined areas protected from the immune response of the host. Beyond, cytoskeletal remodeling and cell-cell contacts are a prerequisite for all routes of virus transmission as interference with both actin and tubulin polymerization strongly reduces HTLV-1 transmission [57,60].

### 4.1. Transmission at Tight Cell-Cell Contacts

#### 4.1.1. Polarized Budding at the Virological Synapse (VS)

**Figure 1:**
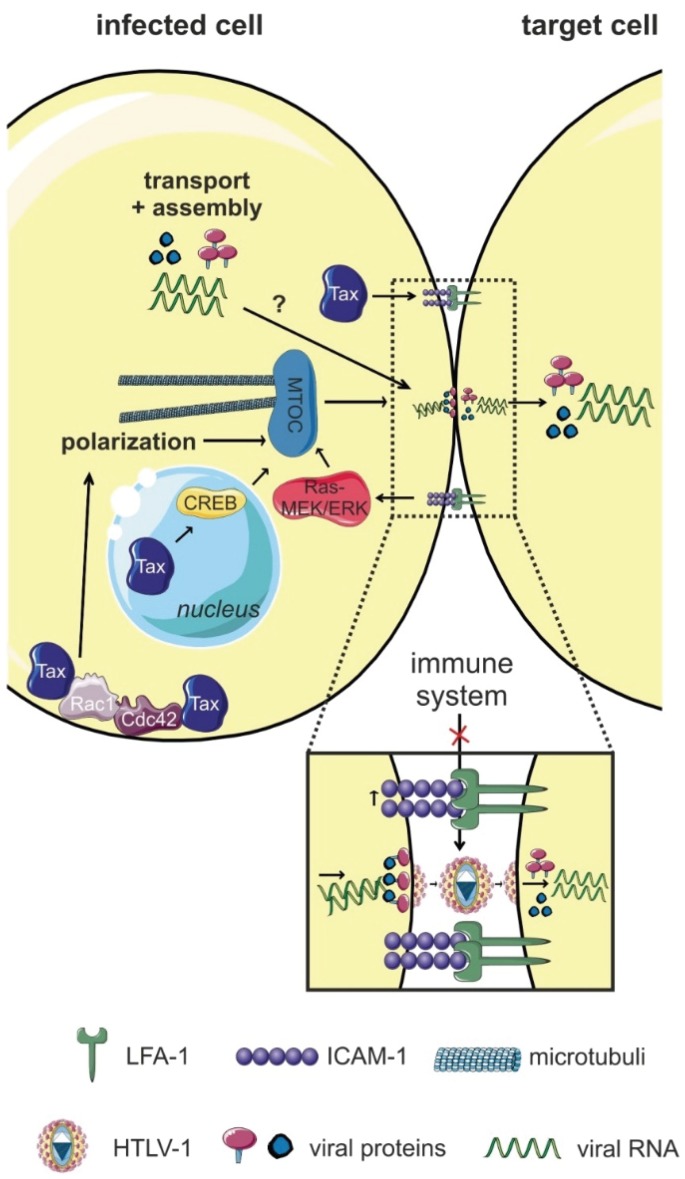
The virological synapse (VS). Interactions of intercellular adhesion molecule 1 (ICAM-1; on HTLV-1-infected T-cells) with lymphocyte function-associated antigen (LFA-1; on target cells), and signals induced by the viral Tax protein trigger polarization of the microtubule organizing center (MTOC) towards the cell-cell contact and formation of the VS at the cell-cell contact. Tax is not only located in the nucleus, but also at the MTOC and in the cell-cell contact region. Tax-induced CREB signaling (nuclear activity of Tax), the accumulation of Tax at the MTOC, and ICAM-1-induced Ras/MEK/ERK signaling are important for MTOC polarization. It is assumed that the VS allows for efficient polarized budding and virus transmission via a synaptic cleft, thus, avoiding recognition of HTLV-1 by the host immune system. Figure was realized thanks to *Servier Medical Art*.

Imaging analysis revealed that HTLV-1 is transmitted from cell-to-cell at the so-called virological synapse (VS; Figure 1) [60]. The VS is defined as a “virus-induced, specialized area of cell-cell contact that promotes the directed transmission of the virus between cells” [61].

Igakura *et al.* found that HTLV-1 Gag p19, Env, Gag p15 (nucleocapsid, important for incorporation of the viral RNA into the particle), and viral genomes accumulate at the interface between primary HTLV-1-infected and uninfected T-cells, followed by viral transfer to the uninfected cell [60]. This transfer was accompanied by polarization of the microtubule organizing center (MTOC) inside the infected cell towards the target cell. The cytoskeletal protein talin, which is important for cell adhesion, also accumulated at this specialized cell-cell contact, and inhibition of actin and tubulin polymerization diminished MTOC polarization [60]. The VS is distinct from the immunologic synapse (IS): contrary to the IS, where the cytoskeleton of the target cell polarizes towards the cell-cell contact, at the VS, the polarization of the cytoskeleton occurs inside the infected cell towards the target cell [61]. MTOC polarization and formation of the VS require at least two signals, one provided by the viral Tax protein, the other provided by the cell-cell contact as follows: (1) The presence of Tax located at the MTOC region and the ability of Tax located in the nucleus to stimulate CREB-dependent signaling pathways; and (2) cross-linking of intercellular adhesion molecule 1 (ICAM-1) at the cell-cell contact [62,63]. ICAM-1 binds to LFA-1 (lymphocyte function-associated antigen 1) on uninfected cells [63,64] at the site of the cell-cell contact, and this interaction could contribute to the preferred tropism of HTLV-1 for CD4^+^ T-cells. Use of specific inhibitors revealed that the small GTPases Rac1 and Cdc42 are important for MTOC redistribution [63]. Electron tomography detected that cell membranes of infected and target cells are closely apposed at the VS, but interrupted by clefts. Gag-positive particles were detected inside the synaptic cleft, which resembled virions in size and morphology [65], suggesting that virions are transferred across this cleft to target cells. However, it is still questionable whether these particles were indeed infectious since no Env was detected at the surface of these particles [65].

Summed up, formation of the VS requires Tax to enhance expression of adhesion proteins (ICAM-1) in an HTLV-1-infected T-cell in contact with an uninfected T-cell [60]. After engagement of ICAM-1 on the infected T-cell and LFA-1 on uninfected T-cells, reorganization of the cytoskeleton in the infected cell occurs. Concomitant with polarization of the MTOC adjacent to the VS, viral proteins are concentrated in the center of the VS and surrounded by an outer ring of adhesion proteins [60]. Thereafter, it is assumed that viral particles are assembled and acquire the viral Env as they bud from the infected cell into the synaptic cleft. Upon induction and binding of the HTLV-1 receptor on the uninfected cell, viral particles cross the VS and enter the uninfected cell [61,66].

Interestingly, polarized assembly and transmission at the VS has also been described for other retroviruses like HIV and MLV [67,68,69]. Contrary to HTLV-1, both HIV and MLV can also spread cell-free. However, viral transmission under conditions of direct cell-cell contact is much more efficient [67,69]. Yet, the quantitative contribution of transmission via the VS for retroviral spread remains to be determined due to the lack of specific inhibitors of polarized budding processes. Taken together, transmission via the VS allows directed transmission of HTLV-1 to target cells whilst avoiding recognition by the host’s immune response.

#### 4.1.2. Transmission of Viral Biofilms at the VS

**Figure 2:**
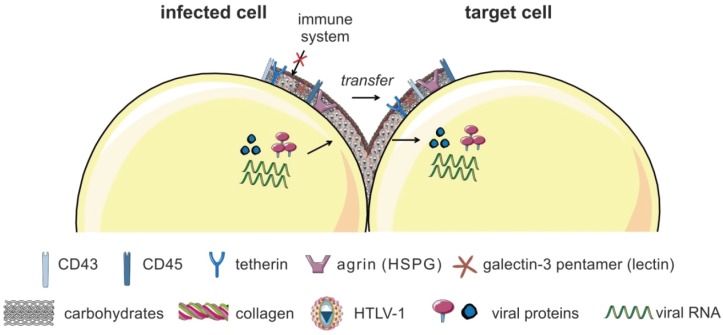
The viral biofilm. HTLV-1 virions are accumulated in a specialized extracellular matrix (ECM), the so-called viral biofilm, on the surface of infected cells. The viral biofilm is composed of carbohydrates, components of the ECM (collagen, agrin), linker proteins (galectin-3, tetherin), and O-glycosylated surface receptors (CD43, CD45). HTLV-1 particles are concentrated into large, highly infectious assemblies that cluster towards the cell-cell contact. HTLV-1 is transferred to target cells and guarded by the biofilm from immune recognition. Figure was realized thanks to *Servier Medical Art*.

After infection of a host, microbes have evolved many issues to be protected from the host immune system. Bacteria developed an important way to hide from the immune system and to spread inside of the host by producing an extracellular biofilm, where bacteria are concentrated outside of infected cells. These distinct environments produced by the microbes themselves are rich in polysaccharides and carbohydrates [70]. Interestingly, biofilms have also been detected on cells infected with HTLV-1 and hence, were named “viral biofilms” (Figure 2) [71]. Biofilm-like, extracellular viral assemblies are composed of extracellular matrix (ECM) components and cellular lectins. In viral biofilms, virions are concentrated in a confined protective environment on the surface of infected cells and are transmitted to target cells at “virological synapses” [71]. HTLV-1 virions and clusters of viral proteins (Gag, Env) are accumulated in this specialized ECM on the surface of cells from infected patients and of chronically-infected cell lines. The biofilm is composed of carbohydrates, components of the ECM like collagen that form tight extracellular matrices, and the HSPG agrin [71]. Additionally, linker proteins (galectin-3, tetherin) [71], and O-glycosylated surface receptors (CD43, CD45) are part of the viral biofilm [72]. Tetherin, which was identified as an antiviral factor, prevents cell-free release of viruses from infected cells, maybe playing a role in the retention of HTLV-1 at the surface of infected cells [73].

HTLV-1 particles are assembled into large, highly infectious clusters and transferred to neighboring cells while being guarded by the biofilm from immune recognition [74]. Treatment with heparin or extensive pipetting removed the viral biofilm and strongly impaired the efficiency of HTLV-1 spreading to target cells by 80% [71], concluding that the viral biofilm is the major contributor of T-cell-associated infectivity. However, the involvement of polarized budding in biofilm formation is not excluded. Compared to the observations at the VS before [60], viral biofilms overlap cell-cell contacts and bridge the gap between both cell surfaces, rather than filling contact sites [71]. Thus, HTLV-1 transmission may not only occur across synaptic clefts, but also at the periphery of the cell contact [61]. The biofilm might also function as viral reservoir as viruses are highly concentrated within these biofilms in close proximity to their target cells. Additionally, cell-free preparations of viral biofilms infect monocyte-derived DC (MDDC) more efficiently than autologous CD4^+^ T-lymphocytes *in vitro* [50]. The viral biofilm could also both provide a physical protection for the viral Env protein [50,75] and prevent recognition of Env by neutralizing host antibodies [76]. It is assumed, that after infection of new cells, viruses reprogram the protein expression of the host, amongst others, to form the viral biofilms [71,76]. Yet, the relative contribution of individual viral proteins to biofilm formation is not settled [74].

Both MLV and HIV also utilize virus-laden uropods for viral spreading at the VS [77,78]. Briefly, polarization of lymphocytes involves the formation of two distinct poles: (1) the leading edge, which attaches the cell to the substrate allowing directional movement of the cell; and, on the opposite side, (2) the uropod, which is mostly involved in cell-cell interactions [79]. The current model suggests that an infected cell will likely engage target cells to form virological synapses if uropods make the initial contact with the target cell [78]. Uropods contain adhesion molecules, Env-laden virions, and adhere to the receptor-expressing target cells, while the leading edge continues to drive cellular polarization of the migrating cells. Contrary, if the leading edge of a migrating lymphocyte makes the initial contact with a target cell, the leading edge will continue to migrate and bypass the target cells [77,78]. Since HTLV biofilms are found as one large or several smaller clusters of viruses bound to the uropod on isolated infected T-cells [71], the uropod might also participate in the formation of the VS during transmission of HTLV-1.

### 4.2. Transmission via Cellular Conduits

**Figure 3:**
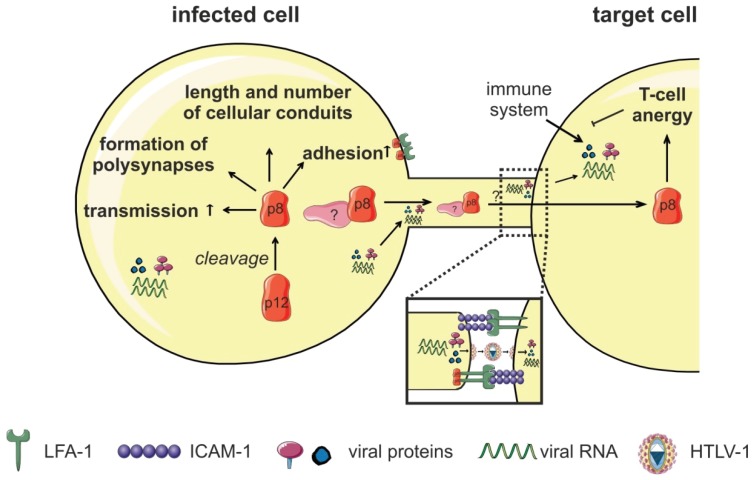
Cellular conduits. The viral accessory protein p12 is proteolytically cleaved into the p8 protein, which increases adhesion of T-cells through lymphocyte function-associated antigen-1 (LFA-1) clustering. Further, p8 induces polysynapse formation and enhances the number and length of cellular conduits between T-cells, thereby, enhancing HTLV-1-transmission. p8 is transferred to target cells through these conduits and it is hypothesized to induce T-cell anergy in the target cell. This might be a strategy for HTLV-1 to evade the host’s immune surveillance during infection. Host cell proteins that interact with p8 to enhance conduit formation, p8 transfer, and HTLV-1 transmission are still unknown. Figure was realized thanks to *Servier Medical Art*.

To allow for transmission of HTLV-1 over long distances, the transfer of virions via cellular conduits induced by the viral p8 protein has been proposed (Figure 3; for details on p8 see Section 6.5). Briefly, p8 is encoded by the open reading frame I of HTLV-1 located in the pX region as a cleavage product of the precursor protein p12 [80]. In co-culture assays with HTLV-1 reporter cells, Van Prooyen and colleagues found that overexpression of p8 rescues the infectivity of p12 knockout molecular clones, and enhances the infectivity of chronically-infected MT-2 cells [58]. Functionally, p8 increased T-cell conjugate formation, potentially through LFA-1 clustering on the surface of T-cells. Surprisingly, overexpression of p8 also enhanced the number and length of cellular conduits among T-cells [58]. Conduits are supposed to be formed by directed outgrowth of a filopodium-like protrusion towards a neighboring cell. In co-cultures between p8-expressing Jurkat T-cells and untransfected Jurkat T-cells, p8 was also detectable in untransfected cells, suggesting transfer of p8 via the conduits. The latter was corroborated by life-cell imaging, which detected fluorescently-labeled Gag and p8 in conduits between chronically-infected T-cells and uninfected target cells. However, it is not known, whether p8 and LFA-1 also cluster at the tip of the conduit, or only at the surface of the infected cell. Finally, transmission electron microscopy showed the presence of viral particles resembling HTLV-1 virions in shape and morphology either at the contact sites between two conduits, or between a conduit and a target T-cell [58]. The authors proposed the model that p8 enhances transmission of HTLV-1 by increasing cellular conduits and polysynapse formation (Figure 3) [58,81].

In parallel, p8 is transferred to neighboring cells, invades target cells and is suggested to induce T-cell anergy by decreasing T-cell receptor (TCR) signaling in target cells, which could favor persistence of HTLV-1 in an immune competent host [58,81]. Taken together, p8-induced virus transmission seems to be a strategy of the virus to be transmitted via long distances. The presence of viral particles at the contact site between conduits and target cells leads to the assumption that HTLV-1 buds from the tip of the conduit towards the target cell via a “mini VS” [58,59]. However, it is not known whether p8 and LFA-1 also cluster at the tip of the conduit, or only at the surface of the infected cell. Formation of a VS between conduit and target cell suggests protected transfer of HTLV between cells and is in contrast to transmission of the related retroviruses HIV and MLV, where isolated viral particles were shown to surf on filopodial bridges before reaching the target cell [82,83]. For HTLV-1, surfing of isolated viral particles has not been observed yet [58]. The detailed molecular mechanism by which p8 promotes HTLV-1 transmission remains unknown. It is conceivable that cellular conduits account for HTLV-1 transmission, as suggested by the authors [58]. Nevertheless, it cannot be excluded that transfer occurs via virological synapses, polysynapses, syncytia, or viralbiofilms [59].

## 5. Cell-Free HTLV-1 Transmission to Dendritic Cells (DC)

**Figure 4:**
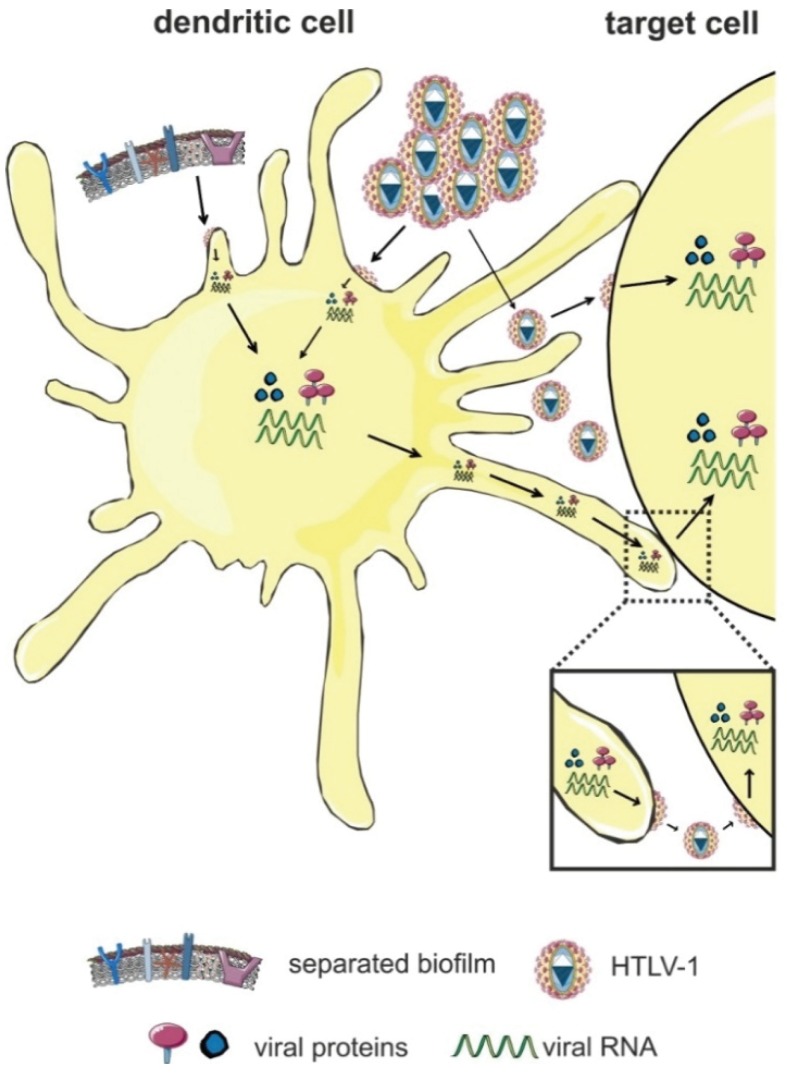
Transmission of HTLV-1 via dendritic cells (DC). DC either capture the virus and transmit it to target cells in the absence of infection (*trans*-infection), or they are productively-infected before viral transmission (*cis*-infection). Productive cell-free infection of DC is achieved *in vitro* by highly-concentrated preparations of cell-free HTLV-1 or by viral biofilms. Figure was realized thanks to *Servier Medical Art*.

Antigen-presenting DC and their precursor cells (monocytes) are found to be infected with HTLV-1 *in vivo* [28,29,30]*.* However, it is not clear whether DC play a role in establishing a chronic HTLV-1 infection. DC either capture virions and transfer them to target cells (*trans*-infection), or they are productively infected and infect other cells themselves (*cis*-infection) (Figure 4) [39,84]. The lectin DC-specific ICAM-3-grabbing nonintegrin (DC-SIGN) facilitates HTLV-1 binding and fusion of DC through an ICAM-dependent mechanism [84,85]. During HIV-transmission, most features previously associated with DC-SIGN-mediated trans-infection of DC are apparently fulfilled by CD169/Siglec-1 [86], whose role remains to be elucidated for HTLV-1.

*In vitro* studies have shown that DC can also be infected cell-free with highly-concentrated viral supernatants, and these infected DC mediate efficient cell-cell contact-dependent infection and transformation of CD4^+^ T-cells [84]. These findings and studies reporting the presence of viral genomes and proteins suggest a potential role of DC in transmission *in vivo* during initial acquisition of infection [61,84]. Interestingly, DC can be infected cell-free via transcytosis through an epithelial barrier [51]. The relevance of DC in viral transmission has been further strengthened by recent findings showing that MDDC are more susceptible to infection with viral biofilms than autologous CD4^+^ T-lymphocytes *in vitro*, which supports the model that infection of DC might be an important step during primary infection *in vivo* [50]. Searching the mechanism, Alais *et al.* found that MDDC express higher amounts of NRP-1 [50], which is part of the HTLV-1 entry receptor [16]. The study also revealed that infection of DC with virus-containing biofilm is much more efficient than infection with concentrated viral supernatants [50]. Thus far, it is not settled whether formation of the viral biofilm is restricted to lymphocytes, or whether it could also be formed upon DC-infection, or whether it could be transmitted via DC-mediated *trans*-infection to other cells. Moreover, infection of DC may also be required for the establishment and maintenance of HTLV-1 infection in primate species [87]. Since the maturation of DC is impaired in HTLV-1-infected patients [88,89], DC may not only contribute to viral dissemination, but also to immune dysregulation observed in HTLV-1-infected patients. Compared to HTLV-1, productive (*cis*) infection of DC with HIV is inefficient due to antiviral mechanisms like the presence of the restriction factor SAMHD1 in DC [86]. Infection of CD4^+^ T-cells occurs in *trans* by DC-captured HIV at the VS [67,90]. It is likely that also HTLV-1 is transmitted from DC to T-cells via polarized budding at the VS, but this has to be verified experimentally.

## 6. Viral Proteins Enhancing HTLV-1 Transmission

Amongst the HTLV-1-encoded proteins contributing to HTLV-1 transmission, we briefly sum up the roles of the structural proteins Env, Gag, and the regulatory protein Rex before we focus on Tax, which is important for formation of the VS (Figure 1) [60], and on p8, which enhances the number of cellular conduits between infected and uninfected T-cells (Figure 3) [58].

### 6.1. Env

Env plays a central role in HTLV-1 cell-to-cell transmission (for review, see [17,40,91,92,93] since Env is crucial for HTLV-1 infectivity. Briefly, Env encodes two different proteins, the transmembrane (TM) and the surface (SU) protein. The precursor protein of Env is highly glycosylated, proteolytically cleaved into SU and TM proteins, and afterwards transported to the cell membrane to initiate virus assembly and budding [17,92]. The SU subunit of Env binds to the host cell surface receptors Glut-1, NRP-1, and to HSPGs to trigger fusion of the membranes both of the virus and the host cell [40]. Env is also important for formation of the VS [66] and for transmission of HTLV-1 *in vitro* and *in vivo* [56,57].

### 6.2. Gag

The HTLV-1 group specific antigen (Gag, p55) is produced as a single precursor polyprotein. Upon posttranslational modification and myristoylation, the Gag polyprotein is targeted to the inner membrane of the cellular plasma membrane [91]. Subsequently, Gag is cleaved by viral proteases into its functional domains matrix (MA, p19), capsid (CA, p24), and nucleocapsid (NC, p15). Matrix is important for Gag targeting, membrane binding, and Env incorporation, while capsid interacts with itself to form the inner core of the virion. Nucleocapsid interacts with the genomic RNA inside the inner core of the virion. A proper spatial and temporal regulation of viral assembly and budding is crucial for HTLV-1 transmission [91,94].

### 6.3. Rex

Among the regulatory proteins, not only Tax, but also Rex is important for viral transmission. This is corroborated by at least two findings: (1) Use of a Rex-deficient HTLV-1 proviral clone showed that Rex is important for viral transmission *in vivo* [91]; (2) The chronically HTLV-1-infected T-cell line C8166-45, which is Rex-deficient, does not produce viral particles, and is not infectious [95]. Taken together, these results suggest that Rex’s function to enhance trafficking of unspliced and single spliced RNA is important for ideal viral spread [91].

### 6.4. Tax

The regulatory protein Tax is essential for viral replication due to strong enhancement of viral mRNA synthesis by transactivating the HTLV-1 LTR (U3R) promoter. Further, Tax is a potent transactivator of cellular transcription and important for initiating oncogenic transformation. Tax shuttles between the nucleus and the cytoplasm and fulfills most of its functions by direct protein-protein interactions [6,19,96,97]. Thus far, not only a plethora of Tax interaction partners [98,99,100], but also of transcriptionally-induced Tax target genes has been identified [101,102,103,104,105]. The latter is attributed to Tax’s function as activator of several signaling pathways including NF-κB, CREB, SRF, PI3K/AKT, and AP-1 [19,106].

Tax is important for HTLV-1 cell-to-cell transmission. First insights were obtained by fluorescent imaging analysis showing that Tax cooperates with ICAM-1 thereby inducing polarization of the MTOC at the VS (Figure 1) [63]. Use of Tax mutants revealed that Tax-induced CREB signaling is critical for MTOC polarization [62]. Interestingly, ICAM-1 is also induced by Tax on the surface of T-cells [107], thus, facilitating the formation of the VS and HTLV-1 transmission. Since engagement of ICAM-1 by interaction with its ligand LFA-1 on target T-cells is important for formation of the VS, Tax-induced ICAM-1 expression may also contribute to the T-cell tropism of HTLV-1 [61]. Use of chemical inhibitors revealed that activity of the small GTPases Cdc42 and Rac1 is critical for Tax-induced MTOC polarization [63]. Since Tax also complexes with these GTPases, Tax might connect Rho GTPases to their targets and affect cytoskeleton organization to favor HTLV-1 transmission [98,99].

Imaging-based methods were pioneering in defining the routes of viral transmission and identifying the localization of viral and cellular proteins involved in transmission. Later, Mazurov *et al.* developed an elegant single-cycle replication-dependent reporter system that allows quantitative evaluation of cell-to-cell transmission by measuring reporter gene expression in newly infected cells [57]. This system requires transient transfection of (1) plasmids carrying a replication-dependent reporter gene; and of (2) virus packaging plasmids. The packaging plasmids encode full-length HTLV-1, or they carry a deletion in the *env* gene and are pseudotyped with VSV-G (glycoprotein G of vesicular stomatitis virus). The reporter plasmids consist of a CMV-driven reporter gene in antisense orientation that is interrupted by a gamma-globin intron in sense orientation. After transcription, the intron is spliced, but the antisense orientation of the reporter gene precludes translation of the reporter mRNAs in transfected cells. These minus strand RNAs are packaged into virions. After infection of new cells, mRNAs are reversely transcribed and reporter gene activity is detectable [57]. Using this system, the authors found that both the cell type and the envelope type are critical for HTLV-1 cell-to-cell transmission: In co-cultures of transfected Jurkat T-cells with Raji/CD4^+^ B-cells, Tax enhanced transmission of HTLV-1 packaged with wildtype Env, but not with HTLV-1 packaged with VSV-G. [57]. On the contrary, the transmission of HTLV-1 reporter vectors in transfected 293T cells was not enhanced by Tax, suggesting that different host factors involved in transmission are induced by Tax in Jurkat T-cells than in 293T cells, possibly due to different signaling pathways being active in the respective cell type. Tax also enhanced cell-to-cell transmission of HIV reporter vectors, suggesting that Tax-induced changes in the infected donor cell are also beneficial for other retroviruses than HTLV-1 [57]. One obstacle when working with these reporter vectors was the lack of sufficient reporter signals in PBMC [57]. Recently, Mazurov and colleagues improved the reporter vectors by modifying the splice sites, and by enhancing packaging efficiency of spliced reporter vectors [108]. It will be interesting to see, which Tax-induced signaling pathways and host factors are required for viral transmission to PBMC.

With regard to pathways important for viral transmission, Tax transcriptionally alters the expression of cell adhesion and surface molecules [109], leading to cytoskeletal remodeling, and complexes with proteins involved in cytoskeleton structure and dynamics [99]. Table 1 lists host factors that are important for HTLV-1-transmission, amongst them are also interaction partners and transcriptional targets of Tax. Despite the knowledge of various Tax-targets involved in cell-cell interaction, adhesion and cytoskeletal organization, a comprehensive analysis evaluating the role of known and new Tax effectors on virus transmission is still lacking. Moreover, it is still not settled whether blocking Tax-induced pathways important for MTOC polarization also impairs cell-to-cell transmission of HTLV-1 reporter vectors.

### 6.5. p8

The HTLV-1 p8 protein is a cleavage product of the viral accessory p12 protein encoded from the open reading frame I. The precursor protein p12 normally localizes to the endoplasmatic reticulum (ER) and to the golgi apparatus, and its functions have been reviewed earlier [21]. p12 is post-translationally modified by a two-step proteolytic cleavage: the first cleavage between amino acid (aa) 9/10 removes an ER-retention signal, which allows trafficking of the protein to the golgi. The second cleavage occurs between aa 29/30 resulting in the p8 protein [80]. p8 is a 70 aa comprising protein that localizes to the cytoplasm and is recruited to lipid rafts and the IS upon TCR ligation [131]. p8 enhances LFA-1-mediated cell adhesion on ICAM-1-coated plates [58]. Earlier work had attributed this function to p12-induced calcium-signaling and suggested that p12 could promote formation of the VS [116] until it became clear that p12 is processed to p8 [58,80]. It has been proposed that p8 enhances HTLV-1 transmission by increasing the number and length of cellular conduits among T-cells (see Figure 3 and Section 4.2). p8-enhanced polysynapse formation and virus transmission from HTLV-1-infected cells to uninfected T-cells [58] had previously been attributed to the precursor p12 [132,133]. Since p8 is also transferred to neighboring cells, invades target cells, and can induce T-cell anergy, it is proposed that p8 favors persistence of HTLV-1 in an immune competent host [58].

Both p8 and p12 form disulfide-linked dimers, and only the monomeric forms of p8 and p12 are palmitoylated at a conserved cysteine residue (C39). Albeit mutation of C39 to alanine abrogates dimerization and palmitoylation, these modifications are dispensable for p8 to increase adhesion and viral transmission [134]. *In vivo* studies in macaques support the notion that p8 and p12 are important for viral persistence and spread. Moreover, productive infection of monocytes depends on the expression of p8 and p12 proteins [87,135]. Cellular effectors and interaction partners of p8 other than LFA-1 that mediate conduit formation, p8-transfer, and viral transmission are still unknown. Interaction partners of p12 have been identified (reviewed by [21,91]), but none of them has been evaluated for a role in p8 transfer and viral transmission. Therefore, the composition of the host machinery that mediates transfer of p8 and HTLV-1 to the target cell remains to be determined.

## 7. Host Factors Involved in HTLV-1 Transmission

HTLV-1 has evolved strategies to manipulate the host cell for its transmission. Not only protein-protein interactions between viral and cellular proteins, but also specific transcriptional induction of host cell factors might facilitate viral transmission. Table 1 lists host proteins, that are involved in HTLV-1 transmission and, if indicated, their manipulation by HTLV-1-encoded proteins. For the sake of completeness, the table also lists proteins which are important for viral entry and syncytium formation.

### 7.1. Cell Surface Receptors and Cell-Cell Contacts

Since cell-cell contacts are a prerequisite for efficient HTLV-1 transmission, it is reasonable that cell surface receptors are critical for this step. Not only receptors on the target cells—like components of the HTLV-1 receptor (Glut-1, NRP-1, HSPGs, SDC-1/-2)—are important for viral transmission and tropism [18,117], but also secreted chemokines that could attract target cells. To attract CCR4^+^CD4^+^ target T-cells, Tax expressing HTLV-1-infected T-cells produce large amounts of CCL22. Expression of CCL2 is stimulated by Tax and block of CCL22 using anti-CCL22 antibodies reduces viral transmission from HTLV-1-infected cells to CD4^+^ T-cells [110].

Although a plethora of surface receptors is upregulated in HTLV-1-infected cells [109], only few of them play a role in virus transmission (Table 1). HTLV-1-induced syncytium formation is affected by Tax, and receptors like vascular cell adhesion molecule 1 (VCAM-1) or ICAM-1 have been shown to promote syncytium formation, and to be inducible by Tax [92,115,118,119,136]. For details about receptors being important for viral entry or syncytium formation, see [17,18,92].

The viral biofilm on the surface of infected cells contains clusters of virions in a cocoon-like structure, and its composition is shown in Figure 2. Thus far, it is not known in detail, whether individual viral proteins are important for biofilm formation. A study by Mazurov *et al.* indicates that large aggregates of HTLV-1 assemblies are more infectious than multiple clustered virions on the surface of infected cells [72]. Their data suggest that heavily O-glycosylated surface receptors CD43 and CD45 render cells less adhesive and prevent inappropriate cell-cell contacts and thus, favor the assembly of HTLV-1 particles into large, highly infectious structures on the surface of T-cells. The authors conclude that a balance between pro- and anti-adhesive molecules on the surface of the infected T-cell is important for the establishment of the VS and virus transmission [72].

### 7.2. Components and Regulators of the Cytoskeleton

Transmission of HTLV-1 and formation of the VS strongly depends on the functional integrity of the cytoskeleton [61]. Experiments using single-cycle replication dependent HTLV-1 reporter vectors confirmed these findings and showed that block of actin and tubulin polymerization strongly reduces HTLV-1 cell-to-cell transmission while transmission of HIV was only modestly impaired [57]. Beyond, Rho GTPases Rac1 and Cdc42, interaction partners of Tax, are involved in MTOC polarization at the VS [63,98]. However, a quantitative comparison of the contribution of individual cytoskeletal proteins and associated regulatory proteins on viral transmission has never been performed.

**Figure 5:**
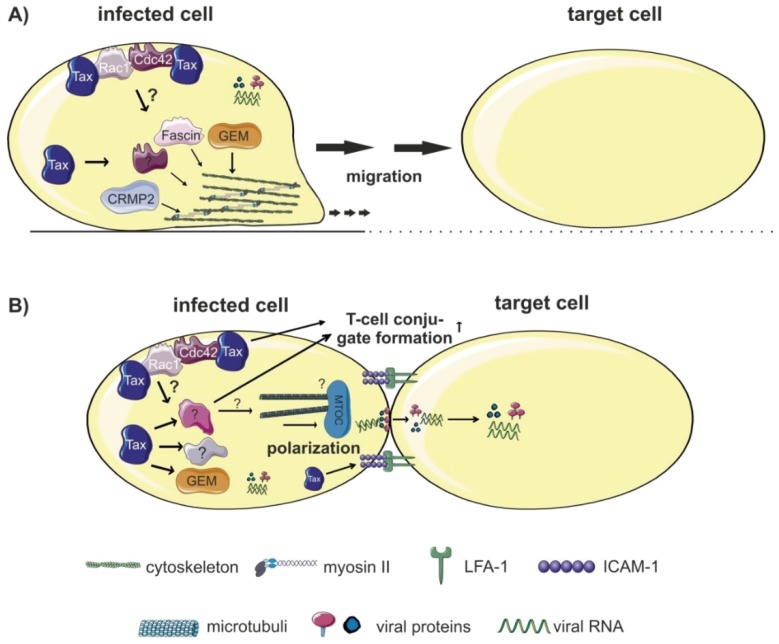
Host factors regulating cellular migration, invasion and conjugate formation. (**A**) Proteins enhancing cellular migration and/or invasion of HTLV-1-infected cells could favor dissemination of HTLV-1 to target cells. Expression of the Tax-induced small GTP-binding protein GEM enhances both migration of HTLV-1-infected cells and viral transmission. Activity of CRMP2, a phosphoprotein involved in cytoskeleton rearrangement, is modulated by Tax and correlates with migration of infected cells. The actin-bundling protein Fascin is induced by Tax and important for invasive migration of HTLV-1-infected cells. A role of CRMP2 and Fascin for viral transmission remains to be determined. Both Rac-1 and Cdc42 are interaction partners of Tax that are crucial for migration and for MTOC polarization. (**B**) T-cell conjugate formation, a prerequisite for cell-to-cell transmission depends on components of the cytoskeleton like the Tax-inducible GEM protein, and on Rac1 and Cdc42. Additionally, Tax regulates expression of surface receptors (see Table 1), which are important for cell-cell contact formation, and, potentially, for formation of the VS and HTLV-1 transmission. The influence of different host factors on polarized budding and formation of the VS remains to be determined. Figure was realized thanks to *Servier Medical Art*.

Host factors regulating cellular migration, invasion and conjugate formation could also be involved in HTLV-1 cell-to-cell transmission by favoring dissemination of infected cells *in vivo* (Figure 5). Among proteins enhancing cellular migration (Figure 5A), the Tax-induced small GTP-binding protein GEM plays an important role in HTLV-1 cell-to-cell transmission [124]. GEM is expressed in HTLV-1-infected T-cell lines and Tax regulates GEM transcription by recruiting CREB and CREB-binding protein (CBP) to the GEM-promoter. Interestingly, GEM is also important for conjugate formation between infected and uninfected T-cells (Figure 5B), which may explain its role in cell-to-cell transmission [124]. However, it is unknown whether GEM and other targets of Tax are required for formation of the VS. The semaphorin-signaling transducer collapsin response mediator protein 2 (CRMP2) has originally been identified in the nervous system where it mediates growth cone navigation induced by semaphorin 3A. Beyond, the phosphoprotein CRMP2 is also involved in cytoskeleton rearrangement controlling migration of human lymphocytes [137]. Activity of CRMP2 is modulated by Tax and correlates with migration of infected cells [120]. It is likely that CRMP2 plays a role in dissemination of infected cells *in vivo* and could thus enhance the probability to transmit viruses to uninfected cells. The actin-bundling protein Fascin is a tumor marker that is highly upregulated in many types of cancer and crucial for invasion and metastasis. We found that Fascin is also important for invasive migration of HTLV-1-infected cells [121]. Fascin is upregulated in chronically HTLV-1-infected T-cells and regulated by Tax through NF-κB signaling [121,123]. Interestingly, CRMP2 and Fascin function downstream of Rho kinases while GEM is an upstream negative regulator of ROCK-I Rho kinase [124]. Currently, we are investigating the role of Fascin in cell-to-cell transmission [122].

### 7.3. Signaling Pathways

Tax is a potent activator of different cellular signaling pathways [19] including CREB, PI3K/AKT, SRF, and NF-κB. However, only little is known about the relative contribution of these signaling pathways on Tax-induced formation of the VS. Using different Tax-mutants, Nejmeddine *et al.* found that CREB signals are important for triggering MTOC polarization, while Ras/MAPK/ERK signals mediate ICAM-1-induced MTOC polarization [62]. Interestingly, expression of the small GTP-binding protein GEM, which has been shown to induce conjugate formation between infected and uninfected T-cells, is also dependent on Tax-induced CREB signaling [124]. However, it remains to be determined whether GEM is involved in MTOC polarization. The contribution of different signaling pathways to formation of the viral biofilm or to p8-induced conduits is not known. It is also not settled whether Jak signaling contributes to p8-mediated virus transmission as has been shown for its precursor p12 [132]. Overall, the quantitative contribution of individual signaling pathways on different mechanisms of viral transmission remains an open question.

## 8. Conclusions

HTLV-1 has evolved several clever strategies to transmit via specialized routes from cell-to-cell, thus being protected from immune recognition. Significant progress has been made in elucidating molecular mechanisms of HTLV-1 cell-to-cell transmission. Nonetheless, the relative contribution of individual pathways on transmission *in vivo* remains to be determined.

## Figures and Tables

**Table 1:** Host cell proteins important for HTLV-1 transmission.

Host Cell Factor	Other Name; Protein Function	Function in Transmission	Modulation by Viral Protein	Reference
***Cell-Surface Associated Proteins***				
Agrin	HSPG; cross-linker of cell surface receptors	biofilm formation		[71]
CCL22	chemokine ligand 22; binding to CCR4	attraction of CCR4^+^ T-cells	induced by Tax	[110]
CCR4	C-C chemokine receptor type 4	on target cell; attracted by CCL22 (from infected cell)		[110]
CD43	leukosialin; sialophorin	adhesion; biofilm formation		[72]
CD45	protein-tyrosine phosphatase	adhesion; biofilm formation		[72]
CD82	Tetraspanin	inhibits syncytium formation	interacts with Gag and Env	[111,112]
Collagen	structural protein of ECM	biofilm formation	induced by Tax (collagen 1 alpha)	[71,113]
DC-SIGN	DC-specific ICAM-3-grabbing nonintegrin	syncytium formation (on target cell DC)		[85]
GLUT-1	glucose transporter 1	virus entry	interacts with Env	[14]
Hsc70	heat shock cognate protein 70	syncytium formation (on target cell)	interacts with Env	[114]
HSPGs	heparan sulfate proteoglycans	virus entry	interact with Env	[16]
ICAM-1	intercellular adhesion molecule 1; CD54	VS formation; MTOC polarization; syncytium formation	induced by Tax	[60,62,107,115]
ICAM-3	intercellular adhesion molecule 3	syncytium formation		[115]
Integrin β2/7	CD18	syncytium formation		[115]
LFA-1	lymphocyte function-associated antigen 1	VS formation (target cell); adhesion (infected cell)	interacts with p8, p12 (infected cell)	[58,60,116]
NRP-1	neuropilin-1	virus entry	interacts with Env	[16]
SDC-1, SDC-2	Syndecan-1/-2; transmembrane HSPGs	virus entry		[117]
Talin	actin-anchor protein; clusters with LFA-1	VS formation		[60]
Tetherin	BST2: bone marrow stromal antigen 2; lipid raft associated protein	biofilm formation; virus attachment		[71,73]
VCAM-1	vascular cell adhesion molecule 1	syncytium formation (on target cell)	induced by Tax (on infected cell)	[115,118,119]
***Cytoskeleton and Associated Factors***				
Actin	structural protein	cytoskeleton remodeling; MTOC polarization; virus release	interacts with Tax	[57,63,98,99]
Cdc42	cell division cycle 42; small GTPase	MTOC polarization	interacts with Tax	[63,98]
CRMP2	collapsin response mediator protein 2	migration, role in transmission unclear	induced by Tax	[120]
FSCN-1	Fascin; actin-bundling protein	invasive migration; cytoskeleton remodeling; cell-to-cell transmission under investigation	induced by Tax	[121,122,123]
***Cytoskeleton and Associated Factors***				
GEM	GTP-binding mitogen-induced T-cell protein	cytoskeleton remodeling; migration; conjugate formation	induced by Tax	[124]
Rac1	Ras-related C3 botulinum toxin substrate 1; small GTPase	MTOC polarization	interacts with Tax	[63,98]
Tubulin	component of microtubule	cytoskeleton remodelling; MTOC polarization		[57,63]
γ-Tubulin	component of centrosomes and spindle pole bodies	cytoskeleton remodelling; MTOC polarization	interacts with Tax	[60,63,99,125]
***Signaling Pathways and Associated Factors***				
CREB	cAMP response element-binding protein	MTOC polarization	interacts with Tax	[62,126]
Jak/Stat	Janus kinase/signal transducer and activator of transcription	syncytium formation		[127]
Ras-Raf-MEK-ERK	rat sarcoma/rat fibrosarcoma/mitogen-activated protein kinase/ERK kinase/extracellular-signal-regulated kinase	MTOC polarization		[62]
***Other Proteins***				
Dlg	disks large homolog	cell-to-cell fusion	interacts with Tax and Env	[128,129]
Galectin-3	beta-galactoside-binding lectin, linker protein	biofilm formation	induced by Tax	[71,130]

cAMP: cyclic adenosine monophosphate; CD: cluster of differentiation; DC: dendritic cell; Env: envelope protein of HTLV-1; ECM: extracellular matrix; GTP: guanosine-5′-triphosphate; MTOC: microtubule organizing center; VLP: virus-like particle; VS: virological synapse.
